# An RPP-YOLOv11 model for road crack detection

**DOI:** 10.1371/journal.pone.0355111

**Published:** 2026-08-06

**Authors:** Yuhong Xue, Ligang Zheng, Yangyang Shi, Jiafeng Bai

**Affiliations:** 1 Shanxi Steel Structure Science & Industry Co., Ltd., Taiyuan, Shanxi, China; 2 School of Journalism and Communication, Xiamen University, Xiamen, Fujian, China; King Mongkut's University of Technology North Bangkok, THAILAND

## Abstract

Accurate and efficient road crack detection serves as a critical component in smart transportation systems and infrastructure maintenance. Existing YOLO series models still exhibit limitations in detecting cracks due to their sensitivity to subtle details, diverse morphological variations, and complex background interference from road surfaces. Building upon YOLOv11, this study enhances road crack detection capabilities through three core innovations: 1) Implementing an early-stage fusion strategy combining visible light and thermal infrared images (RGBT) to improve environmental adaptability; 2) Adopting windmill-shaped convolution modules (PSConv) to replace traditional convolutions, thereby enhancing crack feature extraction while suppressing background noise; 3) Introducing a P6 detection layer to establish a four-scale detection framework (P3-P6), expanding global perception capabilities for large-scale cracks. Experiments on the cross-border road damage dataset RDD2022 demonstrate that the proposed RPP-YOLOv11 model (integrating RGBT multispectral fusion, PSConv convolution modules, and P6 detection layer) achieves 74.90% accuracy, 64.36% recall rate, and 69.04% mAP@0.5 with 42.70% mAP@0.5:0.95. Compared to original YOLOv11 and mainstream benchmarks, this model shows significant improvements in detection precision, robustness, and computational efficiency, providing a reliable technical solution for automated road inspection systems.

## 1. Introduction

As the lifeline of socio-economic operation, the structural health status of road networks is directly related to traffic efficiency and public safety.As the most common early-stage pavement damage, pavement cracks serve as a key indicator for evaluating pavement performance and maintenance requirements [1].If these cracks are not promptly and accurately identified and treated, they will rapidly develop into structural damages such as potholes and web cracks under the combined effects of environmental factors (e.g., water infiltration and temperature fluctuations) and repeated traffic loads. This not only drastically shortens the road’s service life and incurs high maintenance costs but also poses potential safety hazards for vehicle operation [2].Therefore, the development of automated and high-precision road crack detection technology has become a focal point of joint attention in both academic and engineering communities [3].

Traditional detection methods primarily rely on manual visual inspections or detection vehicles equipped with specialized sensors. Manual inspection approaches exhibit inherent drawbacks such as low efficiency, high subjectivity, poor safety, and difficulties in digital data management.While automated inspection vehicles have improved data collection efficiency and standardization to some extent, their high equipment costs, operational expenses, and reliance on fixed routes make it difficult to achieve comprehensive and high-frequency inspections across complex road networks [4].

In recent years, breakthrough advancements in computer vision and deep learning have provided revolutionary technical approaches to overcome the limitations of traditional detection methods [5].In particular, convolutional neural networks and their successful applications in object detection have made image-based automated intelligent disease recognition possible [6].Object detection algorithms can generally be categorized into two types: two-stage approaches (e.g., R-CNN series) and single-stage methods (e.g.,YOLO, SSD) [7].Single-stage detectors represented by YOLO frame the detection task as a unified regression problem, achieving an optimal balance between speed and accuracy. This makes them highly suitable for embedded or real-time processing platforms such as automotive and airborne systems [8].

Since its inception, the YOLO algorithm has undergone rapid and continuous iterative optimization in its architecture.YOLOv5 has been widely adopted due to its exceptional engineering usability [9] and demonstrates outstanding performance in road damage detection tasks [8,10]. YOLOv7 enhances detection accuracy while maintaining lightweight performance through optimized network architecture. The YOLOv7-RDD model specifically improves performance for pavement defect detection [3].YOLOv8 and its variant DBG-YOLO further balance detection accuracy and speed, providing an optimal solution for real-time detection [11]. The lightweight improved version of YOLOv10 demonstrated excellent performance in road crack detection [12].The latest YOLOv11 has undergone systematic optimization in model architecture, training strategies, and inference efficiency. YOLOv11-DCFNet enhances detection capabilities in complex environments through dual-modal fusion [13], while the improved YOLOv11 has been applied to feeder road network defect monitoring [14].

In the field of road crack detection, research on improvements based on YOLO series models has made significant progress.Some studies employ data augmentation techniques to enhance models’ adaptability to complex scenarios [10]. Others optimize network architectures by introducing attention mechanisms such as dynamic snake convolutions to improve crack feature extraction [15]. Additionally, research adopts multi-scale detection strategies, utilizing multi-scale feature aggregation networks to expand model detection capabilities for cracks of varying sizes [16]. To address detection challenges in low-light environments, the LL-YOLO algorithm enhances detection performance under dim lighting conditions through specialized optimization [17]. For small object detection in drone aerial imagery, YOLO-SR achieves enhanced detection accuracy through specialized design [18]. For steel surface defect detection, DSP-YOLO and improved models based on PSConv have also demonstrated promising performance [19,20].

Despite significant progress in existing research, current methods still exhibit multiple limitations: Firstly, most models rely on single visible light image inputs, which leads to substantial performance degradation in complex environments such as low-light conditions, shadows, and haze due to loss of image texture information. Dual-modal fusion methods combining infrared and visible light provide an effective solution to this issue [13,21]. Secondly, road cracks are characterized by small pixel coverage, irregular morphology, and susceptibility to pavement texture interference. Traditional convolutional modules with fixed receptive fields struggle to accurately capture subtle crack features and global semantic information, whereas novel convolutional architectures like windmill convolutions demonstrate advantages in defect detection [19,22]. Thirdly, existing models have limited detection scales, often resulting in segmented missed detection for large-scale targets such as extensive continuous cracks or long-span cracks across lanes, necessitating more comprehensive multi-scale detection systems [16,23].

Multispectral fusion technology provides an effective approach to solve target detection problems in complex environments.RGBT multispectral fusion leverages the complementary advantages of visible light and thermal infrared images, and has been widely applied in all-weather target detection [13,24].The YOLOv11-RGBT framework proposes a single-stage solution for multispectral target detection, demonstrating the feasibility of RGBT fusion [21].Current RGBT fusion strategies primarily include early-stage fusion, mid-stage fusion, and late-stage fusion. Among these, early-stage fusion is more suitable for real-time detection scenarios due to its simple structure and computational efficiency [13].When applying RGBT fusion technology to road crack detection, key challenges such as multi-channel input adaptation and complementary extraction of modal information must be addressed [24].

Convolution module optimization is the core of improving small target detection performance.Traditional standard convolutional receptive fields are symmetric and fixed, making it difficult to accommodate the feature distributions of irregular small targets such as cracks [19].PSConv achieves flexible receptive field expansion and enhanced fine feature capture through asymmetric filling and multi-directional parallel convolution, demonstrating excellent performance in steel defect detection [19,22].The PCMBA-YOLO method, which integrates windmill-shaped convolution with multi-branch auxiliary FPN, demonstrates superior performance in semiconductor laser chip defect detection [22].These studies demonstrate that the design of dedicated convolutional modules is critical for improving small object detection performance.

Refining multi-scale detection architectures is crucial for improving large-scale object detection performance. Current YOLO series models typically employ the P3-P5 three-scale detection framework with a maximum downsample factor of 32, which fails to fully capture the global semantic information of large-scale cracks.HPSM-YOLO significantly improves pavement defect detection performance through a multi-scale feature aggregation network [16].CrackYOLO enhances detection capabilities through multi-scale design for complex rural road scenarios [23].By adding higher-level detection layers, the model’s detection scale range can be expanded, thereby enhancing its ability to recognize large-scale targets [16].

To address the limitations of existing road crack detection models in complex environmental adaptability, fine feature capture, and multi-scale coverage, this study proposes an improved model RPP-YOLOv11 based on the latest YOLOv11 architecture [13,14]. The proposed model integrates RGBT multispectral data, PSConv modules, and P6 detection layers.

The main contributions of this article are as follows:

1. Proposes an RGBT multispectral early fusion strategy that combines RGB and thermal infrared images into a 4-channel input. Through an adaptation module, effective extraction of multimodal information is ensured, significantly enhancing the model’s detection robustness in complex environments such as low-light conditions, shadows, and haze.
2. The PSConv windmill-shaped convolution module is introduced to replace standard convolutions in the backbone network. By employing asymmetric padding and multi-directional parallel convolution, it expands the receptive field to enhance the capture of subtle crack features while suppressing background interference such as road surface textures, achieving a balance between detection accuracy and real-time performance.
3. The newly added P6 detection layer establishes a P3-P6 four-scale detection system, which achieves cross-scale feature fusion through a bidirectional feature pyramid network to enhance global semantic understanding of large-scale fractures and prevent segmented missed detection.

## 2. Preparation work

### 2.1. YOLO series models

The YOLO single-stage detection family, first proposed in 2016, has iterated rapidly for real-time vision tasks. Early versions YOLOv1–v8 completed core foundational innovations: YOLOv1 established regression-based detection pipelines; YOLOv2–v4 optimized anchor boxes, Darknet backbones and data augmentation; YOLOv5–v8 simplified deployment, adopted anchor-free detection and lightweight FPN to balance speed and accuracy, with several variants customized for single-modal pavement crack identification [3,8,10,11]. All YOLOv1–v8 models only support standard 3-channel RGB input and lack dedicated multimodal channel adaptation modules for thermal infrared fusion.

As the latest baseline, YOLOv11 delivers architecture optimizations highly relevant to our all-weather crack detection task: it replaces heavy C2f blocks with lightweight C3k2 modules, introduces a concise SPPELAN neck to cut redundant feature computation, and applies decoupled detection heads with dynamic label assignment to strengthen small crack detection sensitivity. Despite these advantages, vanilla YOLOv11 cannot directly process concatenated 4-channel RGB-T multimodal data, lacking a lightweight input calibration pipeline to unify multimodal channel dimensions.

Two closely related YOLOv11 multimodal variants for road defect detection are analyzed for comparison:

YOLOv11-DCFNet: It implements cross-modal fusion at high-level Neck layers via dual-cross attention, discarding fine-grained low-light crack textures lost in early multimodal features;

YOLOv11-RGBT: It merely adds a single unconstrained 1 × 1 convolution to shrink 4-channel input to 3 channels, with no dedicated noise suppression for infrared background, introducing modal interference into backbone extraction.

Distinct from the two above schemes, our RPP-YOLOv11 designs cascaded Silence and SilenceChannel modules at the input stage for adaptive 4-to-3 channel conversion, which retains valid RGB-T complementary information while filtering thermal noise (detailed in Section 3.1). We further embed pinwheel-shaped PSConv and expand the P6 large-scale detection layer to enhance continuous crack feature learning. Compared with YOLOv11-DCFNet and YOLOv11-RGBT, our early-stage calibrated multimodal fusion achieves superior all-weather detection robustness without obvious inference delay, forming the core novelty of this work.

### 2.2. Feature extraction

Feature extraction serves as the core component in object detection tasks, where its performance directly determines the model’s detection accuracy and robustness [19].The core objective of feature extraction is to extract discriminative visual features from raw images, including low-level features (e.g.,edges and textures), intermediate features (e.g., local shapes), and high-level features (e.g.,global semantics), to provide effective support for subsequent object localization and classification.In road crack detection tasks, crack targets exhibit characteristics such as low pixel coverage, irregular morphology, minimal grayscale variation, and susceptibility to pavement texture and environmental interference, which impose higher demands on the precision, interference resistance, and efficiency of feature extraction modules [23].

Traditional handcrafted design feature operators exhibit limited expressive capabilities, making it challenging to accommodate the complex morphology and dynamic environments of road cracks. Deep learning has driven innovations in feature extraction techniques, with convolutional neural network-based automatic feature extraction methods becoming the mainstream approach.Common backbone architectures include VGG, ResNet, Darknet, and EfficientNet, each balancing feature extraction capability with computational efficiency through distinct structural designs [25].

To enhance feature extraction capabilities for specific targets, the attention mechanism assigns varying weights to feature maps, thereby amplifying target features while suppressing background interference.Novel attention mechanisms such as dynamic snake convolution demonstrate advantages in road damage detection, effectively capturing features of slender targets [15].The PSConv architecture employs asymmetric padding and multi-directional parallel convolution to precisely match the feature distributions of irregular targets such as cracks, effectively enhancing fine feature capture while suppressing background noise [19,22].This module has demonstrated excellent performance in steel defect detection tasks [19], providing a reference framework for road crack detection.The PCMBA-YOLO architecture integrates windmill-shaped convolution with multi-branch auxiliary FPN, significantly enhancing defect detection accuracy [22].ASPCCNet, based on an enhanced ShuffleNet architecture, provides a lightweight solution for pavement crack classification [26].

### 2.3. Feature fusion

Feature fusion is a critical component in object detection, with the core objective of integrating feature information from different levels, scales, or modalities to compensate for the expressive limitations of single features and enhance the model’s ability to detect complex objects [16].In road crack detection, crack targets exhibit multi-scale distribution and diverse feature hierarchies. Single-scale or hierarchical features alone cannot fully characterize the targets, necessitating effective feature fusion strategies to integrate multidimensional feature information.

Feature fusion can be classified into early fusion, intermediate fusion, and late fusion based on the fusion hierarchy [13].Early fusion is performed at the input layer or low-level feature layer, preserving original feature details with high computational efficiency, making it suitable for multimodal data fusion in real-time detection scenarios. Based on feature types, feature fusion can be categorized into intra-modal feature fusion and cross-modal feature fusion.Single-modal feature fusion integrates features from different scales or levels through architectures such as FPN and BiFPN, thereby enhancing multi-scale object detection capabilities [16]; cross-modal feature fusion combines complementary information from different modalities to improve model robustness in complex environments [13,21].

In multimodal fusion, YOLOv11-DCFNet proposes a robust dual-modal fusion method that effectively combines the advantages of infrared and visible light images, thereby improving road crack detection performance in low-light or dark environments [13].The YOLOv11-RGBT framework establishes a unified single-stage architecture for multispectral target detection, offering a novel solution for RGBT fusion [21]. Research on infrared thermal imaging face detection methods also provides technical references for multispectral target detection [24].

In multi-scale feature fusion, HPSM-YOLO achieves effective detection of pavement defects across different dimensions through a multi-scale feature aggregation network [16]. CrackYOLO enhances detection capabilities through multi-scale design for complex rural road scenarios [23]. EPDD-YOLO, based on the Mamba-YOLO architecture, provides an efficient benchmark model for pavement damage detection [2]. Existing YOLO series models typically employ the P3-P5 three-scale detection framework, which struggles to fully capture global semantic information of large-scale targets.Introducing higher-level detection layers and expanding the detection scale range have proven to be effective approaches for enhancing large-scale object detection performance [16]. This study employs an RGBT multispectral early fusion strategy [13,21] combined with the P3-P6 four-scale detection framework and BiFPN architecture [16] to achieve deep cross-modal and cross-scale feature integration.

## 3. Model improvement

### 3.1. Model introduction

Building upon the newly released YOLOv11 architecture, RPP-YOLOv11 enhances road crack detection robustness, multi-scale adaptability, and all-weather performance through systematic improvements across three dimensions: input fusion strategies, feature extraction mechanisms, and multi-scale detection architectures. The model integrates RGBT multispectral data fusion, PSConv windmill-shaped convolution modules, and an enhanced P6 detection layer, establishing a high-performance detection system tailored for complex lighting conditions and multi-scale crack scenarios.

The model adopts an end-to-end processing architecture, sequentially completing four stages: multimodal data input, deep feature extraction, multi-scale information fusion, and object detection and localization. Each module works in close collaboration to establish a complete technical pipeline from dual-spectral image input to crack detection box output. The specific implementation process is as follows:

Input Layer: Early fusion is performed using visible light (RGB) and thermal infrared (T) images. During data preprocessing, RGB three-channel images are spliced with single-channel thermal infrared images to form a four-channel tensor for input into the model. Sequential Silence and SilenceChannel modules are inserted immediately after the 4-channel concatenation to resolve the dimension conflict between multimodal input and the 3-channel YOLOv11 backbone. The Silence module suppresses useless thermal background noise via soft channel masking on the 4-channel tensor, and the subsequent SilenceChannel module adopts learnable 1 × 1 convolution projection to compress the four multimodal channels into three calibrated feature channels; this two lightweight operations output standard 3-channel feature maps compatible with the original backbone without modifying pre-trained weights. This fusion approach preserves complementary information between the two modalities at lower-level features, thereby enhancing the model’s detection capabilities in complex environments such as low-light conditions, shadows, and haze.

Backbone Network: The PSConv (Windmill Convolution) module replaces traditional standard convolutional layers. By employing asymmetric padding and multi-directional parallel convolution, PSConv expands the receptive field to enhance crack feature detection while effectively suppressing interference from road surface textures and background noise. This module maintains lightweight architecture while extracting crack details, achieving optimal balance between detection accuracy and real-time performance.

Neck and Detection Head: A new P6 detection layer (with a downsample factor of 64) has been introduced, which, together with the existing P3–P5 layers, forms a four-scale detection system (P3–P6). The P6 layer utilizes a bidirectional feature pyramid network (BiFPN) to achieve cross-scale fusion with shallow-level features, enhancing the model’s global semantic understanding of large-scale and continuous cracks. This effectively prevents segmented missed detections caused by excessive object scales.

Output and Optimization: The detection head outputs prediction results at four scales: P3 (small cracks), P4 (medium cracks), P5 (large cracks), and P6 (large-scale cracks). By optimizing model parameters through end-to-end training, high-precision localization and classification of full-scale cracks are achieved.

**Fig 1:**
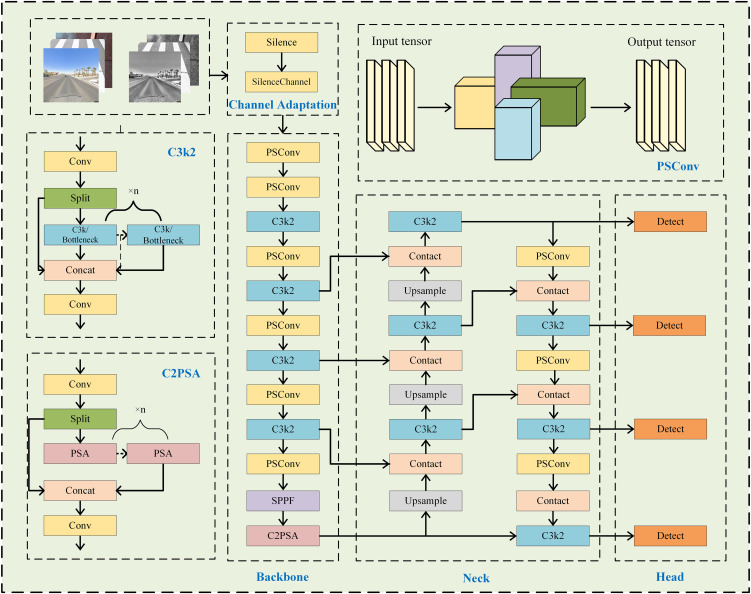
Structural diagram of the RPP-YOLOv11 model.

The structural diagram of the model clearly illustrates the interconnections between modules and data flow patterns, as shown in Fig 1.

The model enhances environmental adaptability through RGBT fusion, improves feature extraction capability via PSConv, and expands detection scale range with P6 layers. While maintaining the efficient inference characteristics of YOLOv11, it significantly improves crack detection performance and robustness in complex road surface scenarios, making it suitable for various practical applications such as drone inspection and vehicle-mounted detection.

### 3.2. RGBT multispectral detection

RGBT multispectral detection is a technology that simultaneously utilizes visible light (RGB) and thermal infrared (TIR) spectral information for target recognition and localization. Visible light images provide rich texture and color information, making them suitable for daytime or well-lit scenarios. In contrast, thermal infrared images capture thermal radiation differences on object surfaces, enabling effective target identification in complex environments such as nighttime, low-light conditions, haze, or shadow coverage. These two modalities exhibit inherent complementarity: visible light demonstrates superior performance under optimal illumination, while infrared excels in low-light conditions or environments with thermal contrast.

In road crack detection tasks, RGBT fusion significantly enhances detection robustness and all-weather adaptability. For instance: During daytime scenarios, visible light images clearly reveal crack textures while infrared images assist in identifying temperature anomaly zones (e.g., temperature fluctuations caused by water infiltration). In nighttime or low-light conditions where visible light quality degrades, infrared images still enable reliable detection through thermal contrast between pavement surfaces and cracks. For complex environments with interference factors like shadows, water accumulation, or stains, dual-modal information can mutually compensate to reduce false positives and missed detections.

**Fig 2:**
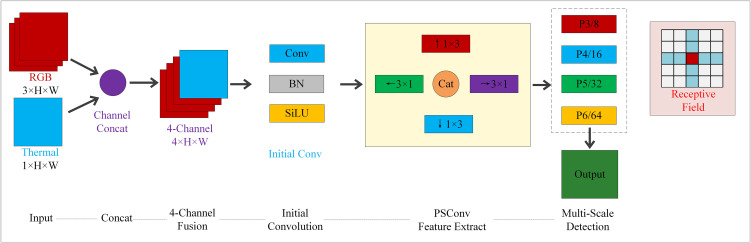
Early fusion of YOLOv11 and RGBT.

Therefore, we adopt the Early Fusion strategy as shown in Fig 2, where visible light (RGB) and thermal infrared (T) images are fused at the input layer. Specifically, the RGB image (3 channels) and thermal infrared image (1 channel) are concatenated into a 4-channel tensor during data preprocessing to serve as model input. This fused tensor is then fed directly into the improved YOLOv11 backbone network for end-to-end feature extraction and object detection.

In the model configuration, we set ch:4 to accommodate 4-channel inputs and utilize the Silence and SilenceChannel modules to adapt input channels, ensuring seamless integration of multimodal data into subsequent feature extraction layers. This fusion approach preserves complementary information between the two modalities at lower feature levels while avoiding complex multi-branch network architectures, thereby facilitating efficient multimodal object detection.

At the mathematical computation level, assuming that $I_{RGB}\in {ℜ}^{H\times W\times 3}$ is visible light image and $I_{T}\in {ℜ}^{H\times W\times 1}$is thermal infrared image, the early fusion result of the 4-channel input tensor $I_{RGBT}$ can be expressed as:


$$\begin{array}{c} I_{RGBT}=Concat\left(I_{RGB},I_{T}\right)\in {ℜ}^{H\times W\times 4} \end{array}$$

Here, denotes Concat(·) the stitching operation along the channel dimension, where H and W represent the image’s height and width respectively.

The merged 4-channel tensor undergoes initial feature extraction through a convolutional layer, with the convolution operation formula as follows:


$$\begin{array}{c} F_{\text{0}}=\sigma \left(BN\left(W*I_{RGBT}+b\right)\right) \end{array}$$

Here, W denotes the weight of the convolution kernel, * indicates the convolution operation, b represents the bias term, BN stands for batch normalization, andσ denotes the activation function (e.g., SiLU).

The subsequent feature extraction process follows the same workflow as the standard YOLOv11 network, employing the C3k2 module and PSConv module for deep feature extraction.


$$\begin{array}{c} F_{i}=C3k2\left(F_{i-1}\right),\text{i}=1,2,\ldots ,5 \end{array}$$

$$\begin{array}{c} P_{\text{j}}=PSConv\left(F_{j}\right),\text{j}=3,4,5,6 \end{array}$$

Here, $F_{\text{i}}$ denotes the feature map of the i-th layer, and $P_{\text{j}}$ represents the feature map output by the j-th detection layer. Through early fusion strategy, the model integrates visible light and infrared information during the input stage, enabling subsequent layers to leverage the complementary characteristics of dual modalities for feature extraction. This approach significantly enhances crack detection performance in complex environments.

This early fusion strategy demonstrates significant advantages in road crack detection: The RGB channels provide high-resolution texture details, facilitating the identification of subtle cracks; the T channel delivers thermal radiation information with strong robustness to lighting variations, effectively addressing challenging conditions such as shadows and nighttime environments. Through synergistic interaction, the model maintains real-time detection capabilities while achieving substantial improvements in crack detection accuracy across all weather conditions and scenarios.

### 3.3. PSConv convolution

**Fig 3:**
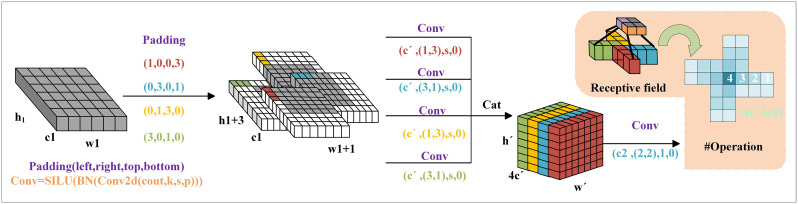
PSConv convolutional architecture diagram.

In road crack detection tasks, crack targets typically exhibit characteristics such as low pixel coverage, irregular morphology, and susceptibility to interference from pavement textures, lighting variations, and stains. Traditional detection methods struggle to balance accuracy and real-time performance. To address this, we introduce PSConv (windmill convolution) combined with the newly added P6 feature layer from the YOLOv11 model to construct an efficient crack detection architecture. As an innovative convolutional structure optimized for small targets, windmill convolution leverages asymmetric filling patterns and multi-directional parallel convolution operations to precisely match the pixel distribution characteristics of road cracks. By moderately increasing parameter quantities, it significantly expands the visual field of view, enhances contrast between cracks and complex pavement backgrounds, and effectively suppresses interference from pavement textures and stains. The computational mechanism involves applying differential filling of 3 pixels or 1 pixel in four directions (left/right/up/down) to input images. Parallel convolution operations using 1 × 3 and 3 × 1 convolution kernels are performed, followed by batch normalization (BN) and SiLU activation functions after each convolution round. The four-direction convolution results are then stitched together, normalized through 2 × 2 convolution kernels, and output as feature maps that ensure both feature extraction integrity and computational efficiency, as illustrated in Fig 3.

In the improved architecture of YOLOv11, PSConv replaces standard convolutions in the lower layers of the backbone network, forming a synergistic optimization with the newly added P6 feature layer. The backbone network sequentially extracts crack features through PSConv layers P1-P5. After optimization via the SPPF module and C2PSA attention module, downsampling via PSConv generates the P6/64 feature layer, which captures more global road surface semantic information and compensates for the loss of small object information in deep features. During subsequent feature fusion, PSConv continuously enhances details through cross-scale integration across P3-P6 layers, working in tandem with the bidirectional feature pyramid network (BiFPN) to achieve bidirectional feature transfer from low-level details (P2) to global semantics (P6), thereby further strengthening feature representation of cracks at different scales.

In mathematical computation, assuming the input road image dimensions are $H_{\text{1}}\times W_{\text{1}}\times C_{\text{1}}$ ($H_{\text{1}}$, $W_{\text{1}}$, $C_{\text{1}}$ representing height, width, and number of channels respectively), the four-direction parallel convolution formula for PSConv is:


$$\begin{array}{c} X_{\text{1}}^{\left({\text{h}}^{\text{'}},\omega^{\text{'}},c^{\text{'}}\right)}=SiLU\left\{BN\left[X_{P\left(1,0,0,3\right)}^{\left(h,\omega ,c\right)}⊗W_{\text{1}}^{\left(1,3,c^{\text{'}}\right)}\right]\right\} \end{array}$$

$$\begin{array}{c} X_{\text{2}}^{\left({\text{h}}^{\text{'}},\omega^{\text{'}},c^{\text{'}}\right)}=SiLU\left\{BN\left[X_{P\left(0,3,0,1\right)}^{\left(h,\omega ,c\right)}⊗W_{\text{2}}^{\left(3,1,c^{\text{'}}\right)}\right]\right\} \end{array}$$

$$\begin{array}{c} X_{\text{3}}^{\left({\text{h}}^{\text{'}},\omega^{\text{'}},c^{\text{'}}\right)}=SiLU\left\{BN\left[X_{P\left(0,1,3,0\right)}^{\left(h,\omega ,c\right)}⊗W_{\text{3}}^{\left(1,3,c^{\text{'}}\right)}\right]\right\} \end{array}$$

$$\begin{array}{c} X_{\text{4}}^{\left({\text{h}}^{\text{'}},\omega^{\text{'}},c^{\text{'}}\right)}=SiLU\left\{BN\left[X_{P\left(3,0,1,0\right)}^{\left(h,\omega ,c\right)}⊗W_{\text{4}}^{\left(3,1,c^{\text{'}}\right)}\right]\right\} \end{array}$$

The four-dimensional convolution results are stitched (Cat) and normalized to produce the final feature map:


$$\begin{array}{c} X^{\text{'}\left(h^{\text{'}},\omega^{\text{'}},c^{\text{'}}\right)}=Cat\left[X_{\text{1}}^{\left(h^{\text{'}},\omega^{\text{'}},c^{\text{'}}\right)},X_{\text{2}}^{\left(h^{\text{'}},\omega^{\text{'}},c^{\text{'}}\right)},X_{\text{3}}^{\left(h^{\text{'}},\omega^{\text{'}},c^{\text{'}}\right)},X_{\text{4}}^{\left(h^{\text{'}},\omega^{\text{'}},c^{\text{'}}\right)}\right] \end{array}$$

$$\begin{array}{c} Y^{\left(H_{\text{1}},W_{\text{1}},C_{\text{1}}\right)}=SiLU\left\{BN\left[X^{\text{'}\left(h^{\text{'}},\omega^{\text{'}},c^{\text{'}}\right)}⊗W^{\left(2,2,C_{\text{1}}\right)}\right]\right\} \end{array}$$

The feature extraction process for the P6 layer is as follows:


$$\begin{array}{c} B\text{ack}bone\left(X\right)=\left\{P_{\text{2}},P_{\text{3}},P_{\text{4}},P_{\text{5}},P_{\text{6}}\right\} \end{array}$$

among


$$\begin{array}{c} P_{\text{2}}=PSConv\left(X\right) \end{array}$$

$$\begin{array}{c} P_{\text{3}}=C3k2\left[Downsample\left(P_{\text{2}}\right)\right] \end{array}$$

$$\begin{array}{c} P_{\text{4}}=C3k2\left[Downsample\left(P_{\text{3}}\right)\right] \end{array}$$

$$\begin{array}{c} P_{\text{5}}=C3k2\left[Downsample\left(P_{\text{4}}\right)\right] \end{array}$$

$$\begin{array}{c} P_{\text{6}}=SPPF\left\{C2PSA\left[Downsample\left(P_{\text{5}}\right)\right]\right\} \end{array}$$

The enhanced architecture demonstrates remarkable advantages in road crack detection: The adaptive receptive field mechanism of PSConv accurately captures detailed features of both microscopic and elongated cracks, while the global semantic information at Layer P6 effectively prevents feature loss in deep networks. These components synergize with the multi-scale feature fusion capability of the BiFPN module, enabling the model to maintain high detection accuracy even in complex road environments. Additionally, PSConv’s lightweight design and optimized network architecture ensure real-time computational performance, meeting demands for real-time detection in scenarios such as drone aerial photography and vehicle-mounted image acquisition. This approach significantly reduces crack detection rates (both missed and false positives), providing reliable technical support for road maintenance decision-making.

### 3.4. P6 detection layer

**Fig 4:**
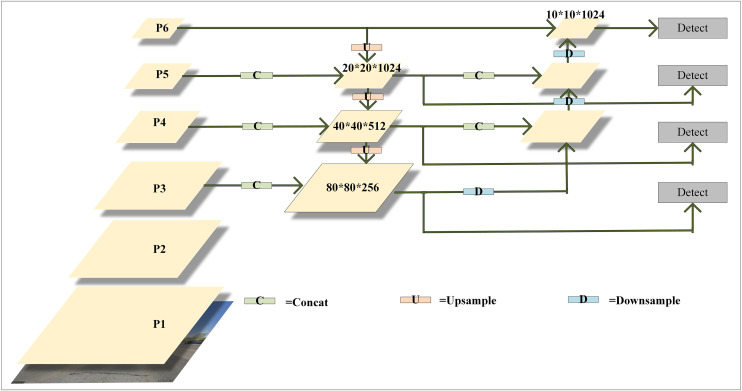
P6 Detection Layer.

In road crack detection tasks, street view images often contain large-scale targets such as extensive continuous cracks and long-span cracks across lanes. These targets occupy wide image areas but exhibit scattered local features, making them susceptible to interference from road debris obstructions and chaotic background textures. Traditional YOLOv11 relies solely on the P3/8, P4/16, and P5/32 three-scale detection framework, which struggles to fully capture global semantic features of large-scale cracks, resulting in insufficient detection accuracy and poor robustness in occluded scenarios. To address this, our improved YOLOv11 model introduces a P6 detection layer (downsample factor 64) as shown in Fig 4. Together with existing scale layers, it forms a “P3-P6” four-scale detection architecture specifically optimized for large-scale crack detection, effectively overcoming challenges posed by severe background clutter and extreme scale diversity. In the backbone network architecture, the input image (4-channel) undergoes preprocessing through the Silence and SilenceChannel modules. Feature extraction across P1-P5 layers is progressively implemented via the PSConv module, with the P5/32 layer maintaining 1024 channels. Subsequently, a 2-step-down sampling operation is executed using the PSConv([1024,3,2]) module to generate the P6/64 feature layer (preserving 1024 channels). This feature layer undergoes feature aggregation through a single SPPF module (5 × 5 convolution kernel), followed by optimization via two C2PSA attention modules. This architecture enhances global correlation of large-scale cracks while suppressing irrelevant background interference, thereby achieving more targeted feature extraction.

In the feature fusion and detection process, the P6 layer employs a bidirectional feature transfer mechanism to achieve multi-scale information complementarity. During the detection head phase, features from the P6 layer are first upsampled to P5 scale via nn.Upsample (nearest neighbor interpolation with scaling factor 2), then concatenated with features from the backbone network’s P5 layer. These features undergo C3k2 module fusion to transfer global semantic features of large-scale cracks to the mid-scale detection layer. Subsequently, P5-layer fused features are further upsampled, sequentially concatenated with P4 and P3 layer features through C3k2 optimization, completing a “top-down” semantic feature transfer. Conversely, P3 layer features undergo PSConv downsampling before merging with P4 layer features, followed by downsampling of P4 features with P5 layer features, and downsampling of P5 features with P6 layer original features. This establishes a “bottom-up” detail feature supplementation mechanism. The Detect module ultimately outputs synchronized detection results across four scales: P3 (small cracks), P4 (medium cracks), P5 (large cracks), and P6 (large-scale cracks).

The P6 layer outputs feature maps with a size of 1/64 of the input image (e.g., 10 × 10 for a 640 × 640 input). With a fixed 1024 channels, this low-resolution design enables efficient global feature extraction while high channel count ensures clear distinction between large-scale cracks and background. This architecture extends the traditional Feature Pyramid Network (FPN) framework, allowing models to extract higher-level semantic information while integrating fine spatial details from lower layers. In road crack detection, it demonstrates significant advantages: capturing complete contour outlines for extensive continuous cracks to avoid segmentation failures caused by large crack spans, accurately identifying crack morphology through deep semantic feature robustness when partially obscured by vehicles or debris, and collaboratively covering the full detection spectrum from micro-cracks to macro-cracks with P3-P5 layers. These enhancements substantially improve accuracy and stability in crack detection within complex urban road environments.

## 4. Experimental results and analysis

### 4.1. dataset

The dataset RDD2022 [27] employed in this study is a large-scale public dataset dedicated to road damage detection, jointly released by multiple research institutions including the University of Tokyo and the Indian Institute of Technology.This dataset is designed to advance the application of computer vision and artificial intelligence technologies in road infrastructure maintenance, particularly for pavement condition monitoring in autonomous driving systems and smart city management. It contains over 47,000 high-resolution road images sourced from multiple countries including Japan, India, and the Czech Republic, covering diverse climate conditions, lighting environments, and road types. Each image is meticulously annotated with eight common road damage categories such as longitudinal cracks, transverse cracks, reticular cracks, and potholes, along with bounding box annotations and pixel-level segmentation labels to support object detection and semantic segmentation tasks. To enhance damage feature representation, we implement binaryization processing analogous to thermal infrared modal analysis, achieving sample-level precise alignment between original RGB images and binaryized images to construct a multimodal visual dataset.

### 4.2. Experimental environment

To verify the performance of the RPP-YOLOv11 model in road crack detection tasks, an algorithm testing framework was constructed using the Python programming language in this study. NVIDIA GeForce GTX 4070 Ti SUPER (16GB graphics memory) was selected as the hardware platform for deep learning training and inference, and the operating system was Windows 11. During the experiments, multi-source images captured by aerial and vehicle-mounted devices for road cracks were cropped and normalized according to their original proportions. All input images were uniformly resized to a four-channel format with a resolution of 640 × 640 pixels to meet the input requirements of the RPP-YOLOv11 model.

In the model training phase, the Adam optimizer was adopted for network parameter optimization. The batch size was set to 32, the total training epoch was 300, and the initial learning rate was set to 0.01. The model was converged and optimized through gradient descent. The experimental dataset was divided into the training set and validation set with a ratio of 7:3, and a reasonable allocation was completed based on the sample distribution characteristics of road crack images, considering the properties of small targets, multi-scale variations, and complex backgrounds in road cracks.

### 4.3. Evaluation indicators

This experiment adopts accuracy ($P=\frac{TP}{TP+FP}$), recall rate ($R=\frac{TP}{TP+FN}$), mean average precision (mAP), and model parameter count (Params) as core evaluation metrics to comprehensively assess model detection performance and lightweight characteristics, aligning with practical road crack detection requirements. Accuracy (P) reflects detection precision by measuring false positive rate, while recall rate (R) indicates detection comprehensiveness by evaluating false negative rate, where TP denotes true positives, FP represents false positives, and FN stands for false negatives. mAP is calculated as the mean area under the precision-recall curve, with experimental focus on mAP@0.5 and mAP@0.5:0.95 to evaluate model performance under conventional and high-precision positioning modes respectivel-serving as the primary benchmark for detection effectiveness. Model parameter count measured in millions (M) reflects lightweight implementation level, directly determining deployment feasibility on resource-constrained platforms such as drones and vehicle-mounted devices.

The coordinated performance of various metrics enables comprehensive model evaluation: accuracy and recall rates ensure low false detection and false omission rates in crack detection, mAP reflects overall detection and localization capabilities, while parameter quantities balance the model’s engineering applicability with real-time inference requirements.

### 4.4. Ablation experiment

To evaluate the performance enhancement of RGBT fusion, PSConv modules, and P6 detection layers on the YOLOv11 model for road crack detection tasks, an ablation experiment was conducted using vanilla YOLOv11 as the baseline model. The experiment set single-module, dual-module combination and triple-module combination groups to progressively verify the contribution of each component, with training and testing performed on the RDD2022 dataset to compare detection performance across all variants. The experimental design and quantitative results are presented in Table 1, where ”√“ indicates the corresponding module is integrated into the network and ” × “ denotes the module is removed.

**Table 1:** Comparison of ablation experimental results for the improved module.

model	RGBT	PSconv	P6	P/%	R/%	mAP@0.5	mAP@0.5:0.95	Params/M
YOLOv11	×	×	×	55.86	43.83	45.19	23.68	2.6
model 1 (YOLOv11 + RGBT)	√	×	×	61.54	50.84	55.37	29.20	2.6
Model 2 (YOLOv11 + PSconv)	×	√	×	62.69	50.97	55.40	30.00	2.5
model 3 (YOLOv11 + P6)	×	×	√	60.55	51.39	55.49	29.49	5.1
Model 4 (YOLOv11 + RGBT+PSConv)	√	√	×	68.27	59.42	64.29	36.30	2.5
Model 5 (YOLOv11 + RGBT+P6)	√	×	√	65.04	57.92	61.80	33.33	4.7
Model 6 (YOLOv11 + PSConv + P6)	×	√	√	67.74	59.42	63.95	36.32	4.5
Ours	√	√	√	74.90	64.36	69.04	42.70	4.1

The results in Table 1 systematically validate the independent effectiveness of each proposed improvement and their mutual synergistic effects in boosting YOLOv11’s road crack detection performance. The baseline YOLOv11 model achieved only 45.19% mAP@0.5, indicating limited detection capability for multi-scale cracks under complex road surface scenarios.

When introducing only one improved component: standalone RGBT fusion elevated mAP@0.5 to 55.37%, demonstrating that multispectral input significantly supplements complementary infrared texture information to improve feature extraction under adverse conditions such as low lighting and shadows. The standalone PSConv module delivered comparable mAP@0.5 of 55.40%, proving its multi-directional asymmetric convolution mechanism enhances fine-grained crack detail capture while effectively suppressing irrelevant pavement background noise. Adding the P6 detection layer alone increased the recall rate to 51.39%, suggesting the expanded large-object detection scale helps reduce missed detections of continuous, wide-range cracks.

For dual-module combined groups: Model4 (YOLOv11 + RGBT+PSConv) obtained a notable mAP@0.5 of 64.29%, which outperforms any single-module variant; the joint use of multimodal input and optimized convolution achieves coordinated promotion of feature quality at the input and backbone stages. Model5 (YOLOv11 + RGBT+P6) reached 61.80% mAP@0.5, revealing that RGBT fusion paired with extended detection layers balances multimodal information utilization and multi-scale coverage. Model6 (YOLOv11 + PSConv + P6) achieved 63.95% mAP@0.5, verifying that feature extraction enhancement and expanded detection scale together alleviate both small crack feature blurring and large crack missing detection problems. All three dual-module combinations achieve obvious performance gains over single-module groups, reflecting preliminary synergy between every pair of improvements.

When all three improvements (RGBT+PSConv + P6) are fully integrated into our proposed RPP-YOLOv11 (Ours row), model performance achieves a significant leap with mAP@0.5 reaching 69.04% — a 23.85 percentage point improvement over the baseline — accompanied by precision and recall rates rising to 74.90% and 64.36%, respectively. This demonstrates strong cumulative synergistic enhancement effects from the three modules: RGBT fusion enriches input multimodal information, PSConv refines discriminative crack feature extraction, and the P6 layer expands the network’s multi-scale detection range. The three components jointly elevate the comprehensive detection capabilities of the model under complex all-weather pavement environments.

**Fig 5:**
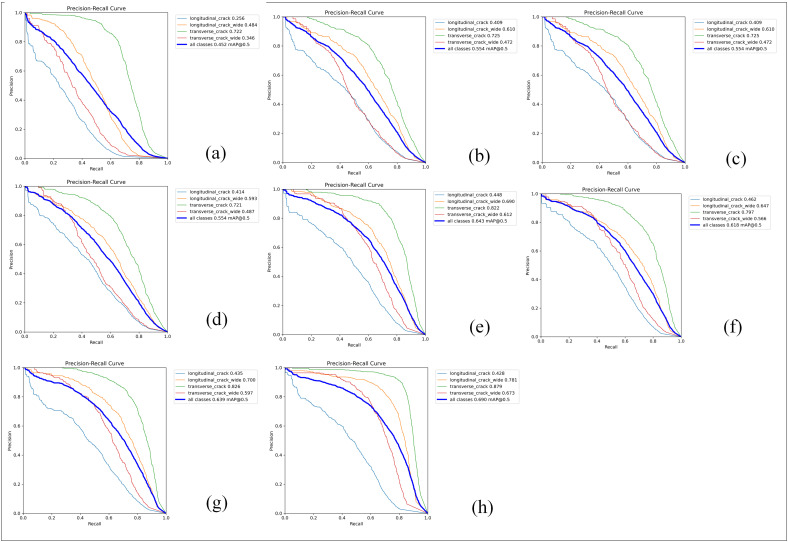
Comparison of PR curves in ablation experiments: (a) PR curve of YOLOv11 model; (b) PR curve of YOLOv11 + RGBT model; (c) PR curve of YOLOv11 + PSConv model; (d) PR curve of YOLOv11 + P6 model; (e) PR curve of YOLOv11 + RGBT+PSConv model; (f) PR curve of YOLOv11 + RGBT+P6 model; (g) PR curve of YOLOv11 + PSConv + P6 model;(h) PR curve of RPP-YOLOv11 model.

Fig 5 visually demonstrates the performance differences among all single-module, dual-module and triple-module combinations through precision-recall curves, with subplots (a)–(h) corresponding to the eight ablation groups in Table 1 respectively. The baseline YOLOv11 model (a) exhibits the lowest overall curve position, particularly showing a sharp precision decline as recall rates increase, indicating severe difficulty in balancing false negatives and false positives for all four crack categories. Introducing single improved modules separately delivers moderate performance gains: the RGBT-only variant (b) and PSConv-only variant (c) both lift the PR curve upward and deliver more stable detection precision across recall intervals; the P6 single-module model (d) yields a smoother curve trajectory within high recall ranges, effectively boosting detection coverage for large transverse wide cracks.

For dual-module combined schemes, obvious coordinated performance improvements can be observed from their PR curves. The YOLOv11 + RGBT+PSConv model (e) achieves a larger enclosed area under the curve than all three single-module groups, as multimodal complementary information and multi-directional feature extraction jointly raise precision for tiny longitudinal cracks. The YOLOv11 + RGBT+P6 model (f) presents balanced curve elevation for both small narrow cracks and large continuous cracks, benefiting from the combined effect of infrared supplementary features and expanded multi-scale detection range. The YOLOv11 + PSConv + P6 model (g) further stabilizes precision at high recall values, where optimized receptive field extraction and extended P6 scale together reduce both missed large cracks and misidentified background noise. All three dual-module curves sit distinctly above every single-module counterpart, verifying preliminary synergies between each pair of proposed components.

The fully improved RPP-YOLOv11 model (h) demonstrates significantly higher PR curves compared to all seven ablation variants, maintaining outstanding precision across the entire recall range and possessing the maximum area under the curve for all crack classes.

In conclusion, the ablation experiment results demonstrate that each improvement module can enhance the model’s detection performance to varying degrees. When used in combination, they generate synergistic effects, further improving the model’s overall performance.

### 4.5. Comparative experiment

To further validate the superiority of the RPP-YOLOv11 model, comparative experiments were conducted with mainstream YOLO series models (YOLOv5, YOLOv8, YOLOv10, and YOLOv11) on the RDD dataset. The results of the comparative experiments are presented in Table 2.

**Table 2:** Comparison of detection performance between the RPP-YOLOv11 model and other advanced methods on the RDD2022 dataset.

method	precision (P/%)	recall (R/%)	mAP@0.5	mAP@0.5:0.95	Parameter quantity (Params/M)
YOLOv5	56.34	42.57	43.69	21.91	2.5
YOLOv8	56.38	44.02	45.30	23.36	3.0
YOLOv10	58.64	43.23	45.60	24.33	2.7
YOLOv11	55.86	43.83	45.19	23.68	2.6
Ours	74.90	64.36	69.04	42.70	4.1

As shown in Table 2, the RPP-YOLOv11 model in this study significantly outperforms YOLOv5, YOLOv8,YOLOv10, and the original YOLOv11 model across all key metrics. The RPP-YOLOv11 model achieves an accuracy rate of 74.90%, surpassing the second-best model YOLOv10 by 16.26 percentage points. Its recall rate reaches 64.36%, far exceeding other models (with a maximum of 44.02%), demonstrating exceptional object detection coverage. In the core evaluation metric mAP, the improved model achieves mAP@0.5 and mAP@0.5:0.95 values of 69.04% and 42.70%, respectively, representing enhancements of over 23 and 19 percentage points compared to the original YOLOv11 model. This indicates stable performance across varying localization accuracy requirements. Despite significant performance improvements, the model maintains lightweight characteristics with only 4.1 million parameters. Overall, the RPP-YOLOv11 model achieves comprehensive enhancements in accuracy, recall, robustness, and efficiency through multispectral information integration, optimized convolutional architecture, and expanded detection scales, providing a high-performance practical solution for road crack detection.

**Fig 6:**
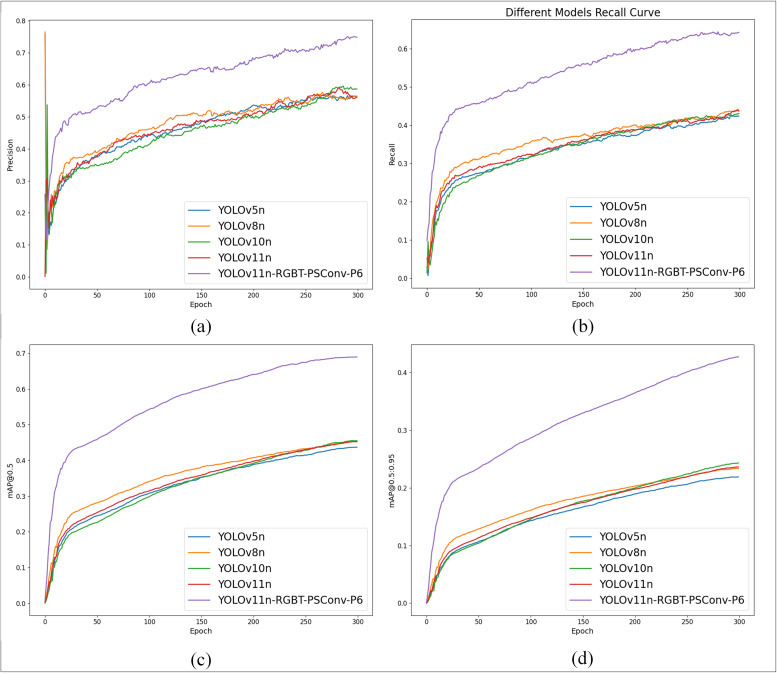
Comparative plots of different models P, R, mAP@0.5, and mAP@0.5:0.95 (a) Precision curve comparison (b) Recall curve comparison (c) mAP@0.5 curve comparison (d) mAP@0.5:0.95 curve comparison.

Fig 6 visually compares the performance of various models across four core metrics through graphical curves. The RPP-YOLOv11 model demonstrates exceptional performance across accuracy, recall, mAP@0.5, and mAP@0.5:0.95, maintaining a clear lead. Its high accuracy reflects low false detection rates, enhanced by RGBT fusion’s environmental information augmentation and PSConv’s feature refinement. The significant improvement in recall indicates low missed detection rates, primarily attributed to the P6 layer’s expanded detection scale range and multi-scale fusion mechanism. The comprehensive superiority in both mAP metrics-particularly the high mAP@0.5:0.95 value-confirms the model’s stable performance under strict localization requirements, validating its refined capabilities in feature extraction and localization regression. This visualization conclusively demonstrates RPP-YOLOv11’s comprehensive advantages in road crack detection tasks.

**Fig 7:**
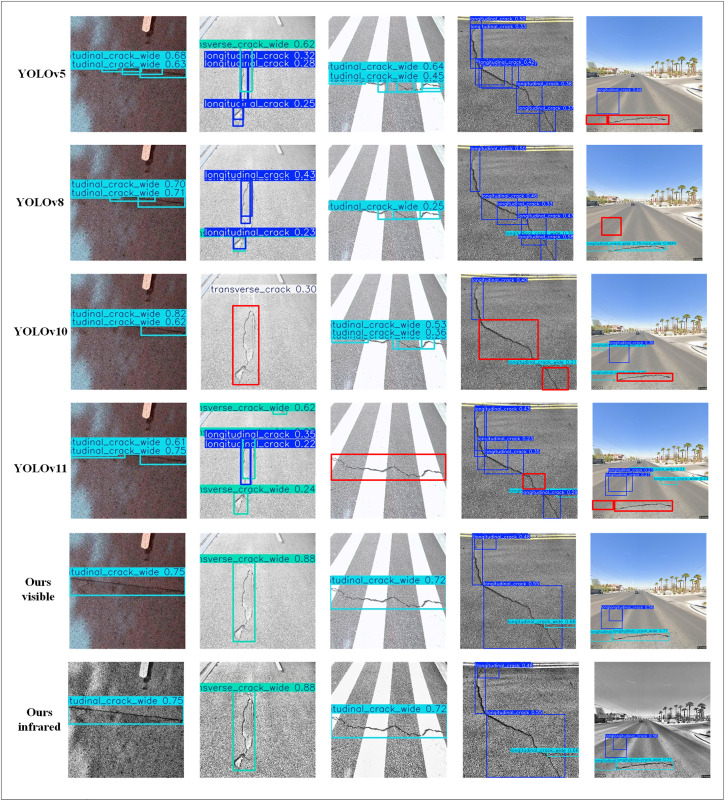
Comparison of detection results across different models.

Fig 7 visually demonstrates the superiority of the RPP-YOLOv11 model through comparative analysis of real-world detection results. When applied to complex road surface images, models like YOLOv5 and YOLOv8 exhibited significant shortcomings including missed detections (particularly for elongated or network-like cracks), false positives (misidentifying textures as cracks), and inaccurate localization. The red-boxed regions clearly highlight each model’s limitations: In the shaded road scenario shown in Figure (a), both YOLOv5 and YOLOv8 failed to detect longitudinal cracks; Figure (b) revealed varying degrees of missed detections or localization errors across YOLOv5, YOLOv8, and YOLOv10 for transverse cracks; Figure (c) demonstrated YOLOv11’s notable oversight of fine cracks in networked crack areas; Figure (d) showed incomplete crack coverage by YOLOv5, YOLOv8, and YOLOv10 in large-scale crack scenarios; and Figure (e) revealed inadequate long-range crack detection capability in distant road sections by YOLOv5 and YOLOv8. In contrast, our RPP-YOLOv11 model exhibits comprehensive and precise detection capabilities: successfully identifying cracks in shaded areas (via RGBT fusion), accurately recognizing fine cracks (through PSConv feature enhancement), and precisely locating large-scale continuous cracks (using P6-layer global perception). Detection bounding boxes closely match crack morphology with minimal false positives. This comparison confirms that our improved strategy significantly enhances the model’s reliability and practicality in real-world complex road environments.

### 4.6. Extension experiment

To comprehensively validate the generalization capability and practical applicability of the proposed RPP-YOLOv11 model, this section conducts dedicated extension experiments. In addition to the RDD2022 road crack dataset used in this study, we incorporate the publicly available and authoritative UAV-PDD2023 road damage dataset [28] as a cross-domain testing benchmark. This dataset, collected through aerial drone imaging, contains road damage images from various complex scenarios with distinct acquisition perspectives (top-down/side-view), imaging heights (30–100 meters), lighting conditions (bright light/shadow/dusk), and crack morphological features (significant scale variations and complex backgrounds) that significantly differ from RDD2022. These variations enable objective evaluation of the model’s cross-scenario adaptability. The extension experiments exclusively use the RPP-YOLOv11 model as the test subject, conducting independent validation on both datasets without introducing comparative models to assess feature transfer capability and robustness.

Table 3 presents the quantitative results of RPP-YOLOv11 model’s extension experiments across two datasets. All detection metrics represent the model’s true performance without fine-tuning, objectively reflecting its generalization capability.

**Table 3:** Extension experiment results of the RPP-YOLOv11 model on different datasets.

Test dataset	precision (P/%)	recall (R/%)	mAP@0.5	mAP@ 0.5:0.95	loss (%)	False positive rate (%)	FPS
This dataset	74.90	64.36	69.04	42.70	35.64	25.1	323.25
UAV-PDD2023 dataset	74.94	54.35	60.64	31.98	45.65	25.06	182.55
Amplitude of indicator change	0.04	−10.01	−8.4	−10.72	10.01	−0.04	−140.7

Table 3 demonstrates that the RPP-YOLOv11 model achieves outstanding performance on the RDD2022 dataset (mAP@0.5: 69.04%). When directly applied to the UAV-PDD2023 dataset, the model exhibits reasonable performance fluctuations but maintains high core metrics: accuracy remains stable at 74.94% with mAP@0.5 of 60.64%—a slight decrease from the source dataset indicating robust basic detection capabilities. However, recall rate declines to 54.35% while false negative rate increases, likely attributable to variations in crack morphology and acquisition angles across datasets. The model’s parameter size and inference speed (182.55 FPS) still meet real-time requirements. Results confirm that the model’s ability to learn multispectral and multi-scale features endows it with strong cross-scenario adaptability, providing generalization assurance for practical engineering deployment.

**Fig 8:**
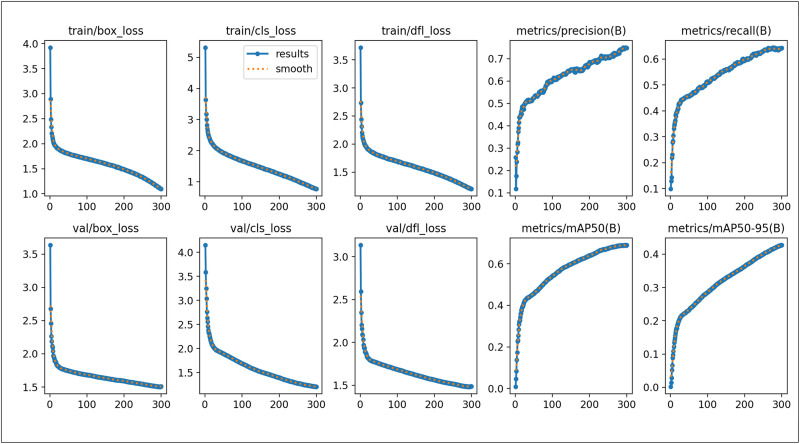
Loss function of RDD dataset, P, R, mAP@0.5, mAP@0.5:0.95 visual comparison.

**Fig 9:**
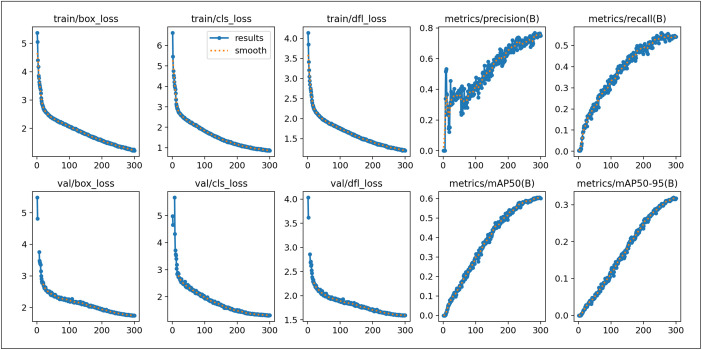
Loss function of the UAV-PDD2023 dataset, P, R, mAP@0.5, mAP@0.5:0.95 visual comparison.

Figs 8 and 9 demonstrate the loss curves and evaluation metrics of the model on RDD2022 (training set) and UAV-PDD2023 (test set). Both loss curves exhibit smooth convergence, indicating stable training performance. The evaluation metrics on the test set show similar trends to those on the training set, with accuracy curves nearly overlapping, demonstrating strong generalization capability in distinguishing cracks from non-cracks. Although recall and mAP curves remain relatively low on the test set, their steady progression confirms the effectiveness of the detection mechanism, with performance variations primarily attributed to data distribution differences. These findings validate RPP-YOLOv11 model’s excellent feature transfer capability from a learning process perspective.

**Fig 10:**
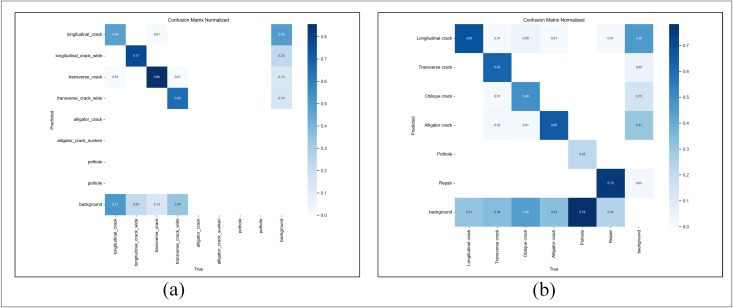
Comparison of confusion matrices across different datasets (a) Confusion matrix on RDD2022 dataset (b) Confusion matrix on UAV-PDD2023 dataset.

Fig 10 evaluates the model’s generalization performance through confusion matrix comparisons at classification granularity. On RDD2022, the model demonstrated accurate classification of “cracks” and “background” with minimal false positives and false negatives. On UAV-PDD2023, the matrix structure remained largely intact while maintaining high background classification accuracy, indicating robust false positive control. However, increased false negatives in crack categories correlated with declining recall rates, likely due to significant feature variations in cracks across the new dataset. Overall, the model avoided severe misclassifications and exhibited robust classification logic, further validating its adaptability for real-world multi-scenario applications.

**Fig 11:**
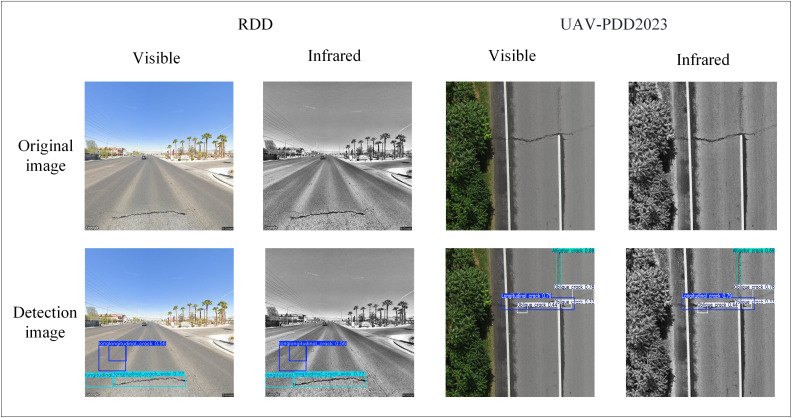
Comparison of detection results across different datasets.

Fig 11 visually demonstrates the practical detection performance of the RPP-YOLOv11 model across two distinct datasets. On the RDD2022 dataset (top), the model achieves precise localization of various crack types (longitudinal, transverse, and reticular) with detection boxes closely matching crack geometries and minimal background interference. On the UAV-PDD2023 dataset (bottom), despite variations in acquisition angles, lighting conditions, and crack morphology, the model maintains robust detection capabilities, successfully identifying most crack targets. Comparative analysis reveals strong cross-dataset generalization performance, with only minor missed detections observed in fine crack details and complex background regions. The overall detection stability is satisfactory, validating the reliability of our proposed improvement strategy for practical engineering applications.

## 5. Conclusion

This study addresses challenges in road crack detection tasks, including poor environmental adaptability, difficulties in extracting subtle features, and insufficient multi-scale coverage, by proposing an RPP-YOLOv11 model. The model significantly enhances detection robustness under adverse conditions such as low lighting, shadows, and haze through early RGBT multispectral fusion strategies. It replaces traditional convolutional layers with PSConv windmill-shaped convolution modules to effectively capture fine crack features while suppressing background noise interference. The addition of a P6 detection layer and establishment of a four-scale detection system (P3-P6) further expand the model’s global perception range for large-scale cracks, avoiding segmented detection gaps. Experimental results on RDD2022 dataset demonstrate that the proposed RPP-YOLOv11 model outperforms original YOLOv11 and mainstream benchmarks in key metrics including accuracy, recall, and mAP, while maintaining low parameter complexity and real-time inference performance. Ablation experiments validate the effectiveness and synergy of improved modules, while comparative experiments confirm superior detection capabilities in complex scenarios. Extended experiments show consistent performance stability across datasets, demonstrating strong generalization ability and engineering applicability.

In conclusion, the RPP-YOLOv11 model proposed in this study provides an efficient, robust, and practical solution for road crack detection, which is expected to play a significant role in intelligent transportation infrastructure maintenance scenarios such as drone inspection and vehicle-mounted detection. Future research will further explore optimized deployment of the model on embedded devices and attempt to integrate more modal information to address more complex road environments.
