# Supplementary material for: An RPP-YOLOv11 model for road crack detection
Source: PLoS One. 2026 Aug 6;21(8):e0355111. doi: 10.1371/journal.pone.0355111 (PMC13446698; doi:10.1371/journal.pone.0355111)
Supplement: Supplementary file 1 [file pone.0355111.s002.docx]

Dear Academic Editor Dr. Ardahir Mohammadzadeh and respected Reviewers,

We sincerely appreciate the valuable comments, detailed suggestions and constructive revisions from the academic editor and two reviewers. We have carefully studied all the feedback, revised the manuscript item by item strictly following your requirements, supplemented missing experimental content, adjusted format specifications, optimized figures and relevant statements. Below is our point-by-point response to all questions and requirements. The revised manuscript with track changes, clean manuscript and all supplementary files are submitted together as required.

**Part 1 Response to General Requirements from PLOS ONE Academic Editor**

**1. Manuscript Format Compliance**

We have fully revised the manuscript in accordance with the PLOS ONE official formatting templates (main body and title & author affiliation templates). All layout, font, paragraph format, citation style and table/figure caption rules have been adjusted to meet the journal’s standard requirements.

**2. Code Sharing Policy**

Following PLOS ONE’s code sharing and reproducibility guidelines, we have uploaded the complete source code of the proposed RPP-YOLOv11 model to a public open-source repository. The access link is: https://github.com/wolfololo/RPP-YOLOv11. The code is fully open for peer review, experimental reproduction and academic reuse, complying with the journal’s code sharing best practices.

**3. Funding Statement Revision**

As required, we have removed all funding-related content from the Acknowledgments section of the manuscript. The revised formal funding statement is provided below for updating in the online submission system:

This research was funded by the Technology Development Cooperation Project between Shanxi Steel Structure Science & Industry Co., Ltd. and North University of China (Development of Digital Twin Intelligent Inspection System for Building Steel Components, Contract No.: 20260320161524). The funders provided salary support for the authors affiliated with Shanxi Steel Structure Science & Industry Co., Ltd., but had no role in the study design, data collection and analysis, decision to publish, or preparation of the manuscript. The specific contributions of all authors are detailed in the Author Contributions section.

**4. Competing Interests & Commercial Relationship Statement**

Some authors are employed by Shanxi Steel Structure Science & Industry Co., Ltd. We hereby submit the updated competing interests statement as follows:

One or more authors are employed by Shanxi Steel Structure Science & Industry Co., Ltd. This commercial affiliation does not alter our adherence to all PLOS ONE policies on sharing data and materials. There are no patents, products in development or marketed products related to this work to declare.

**5. Data Availability & Minimum Dataset Confirmation**

The public datasets for road defect detection used in this study can all be obtained through official designated channels, with the specific acquisition methods as follows:

RDD2022 Dataset：https://github.com/sekilab/RoadDamageDetector

UAV-PDD2023 Dataset：https://doi.org/10.1016/j.dib.2023.109692

The source code of the proposed RPP-YOLOv11 model is available at https://github.com/wolfololo/RPP-YOLOv11 for peer review and experimental reproduction.All minimal datasets required to replicate all experimental results of this study, including model evaluation metrics, training records, raw curve images, confusion matrices and detection visualization figures, are provided within the manuscript’s Supporting Information supplementary file.

**6. Copyright Issues of Figures**

We hereby clarify the copyright of all figures in the manuscript:

Figure 1: model structure diagrams is independently drawn by the authors, with no external copyright restrictions.

Figure 7, Figure 11: These experimental result comparison graphs are generated from our self-conducted road crack detection experiments, using original data and images collected in this study. There is no third-party copyright involved.

All figures have been converted to PDF vector format with a resolution higher than 300 DPI as required. The blurriness in the previous submission was caused by platform automatic compression. The newly uploaded high-resolution PDF figures ensure all text, labels, arrows and graphical elements are clear for both screen display and printing. No copyright permission from third parties is needed for all figures in this manuscript.

**Part 2 Response to Comments from Reviewer 1**

**Comment 1**: Revise Section 2.1: Shorten the overview of YOLOv1–v8, elaborate on YOLOv11’s improvements related to this work, and clearly compare RPP-YOLOv11 with YOLOv11-DCFNet and YOLOv11-RGBT to highlight novelty.

Response:

We have comprehensively revised Section 2.1 YOLO Series Models as required:

We greatly condensed the introductory content of YOLOv1 to YOLOv8, retaining only core functional characteristics closely related to pavement detection and removing redundant iterative descriptions.

We supplemented detailed explanations of YOLOv11’s native architectural optimizations, including lightweight C3k2 modules, SPPELAN neck and decoupled detection heads, and analyzed the defects of the original YOLOv11 in processing 4-channel RGBT multimodal data.

We made a targeted comparative analysis between our RPP-YOLOv11 and two closely related variants (YOLOv11-DCFNet, YOLOv11-RGBT). We pointed out the shortcomings of late fusion and simple channel compression in the existing methods, and highlighted the novelty of our cascaded Silence & SilenceChannel modules, PSConv and P6 detection layer. The revised content has been marked in the track-changes manuscript.

**Comment 2**: Provide high-resolution figures (Figure 1, 2, 3, 4) with clear labels and elements (minimum 300 DPI, vector format such as PDF/SVG is preferred).

Response:

We have reprocessed all key figures. All figures are exported as PDF vector files with a resolution of 300 DPI or above. The blurry problem in the last submission was caused by the platform’s automatic compression of raster images. The newly uploaded PDF figures guarantee that all text labels, arrows, network structures and module details are clearly recognizable on screen and in printed versions.

**Comment 3**: In Section 3.1 (RGBT fusion), briefly explain the core functions of Silence and SilenceChannel modules, as well as their specific operations for 4-channel input to adapt the 3-channel backbone network, to improve readability.

Response:

We have added targeted descriptions of the two modules in the Input Layer part of Section 3.1 on the basis of the original text:

We briefly stated the core function of Silence and SilenceChannel: solving the channel dimension mismatch between 4-channel RGBT input and the original 3-channel YOLOv11 backbone.

We detailed the specific operations: the Silence module suppresses invalid thermal infrared background noise via soft channel masking on the 4-channel tensor; the SilenceChannel module uses learnable 1×1 convolution to compress 4 channels into standard 3-channel feature maps.

We clarified that this lightweight operation does not modify the pre-trained weights of the backbone network, realizing seamless adaptation. The revised content is concise and compliant with the requirements, and the readability of this section is significantly improved.

**Part 3 Response to Comments from Reviewer 2**

**Comment 1**: Poor clarity of all figures and non-standard table layout.

Response:

Figures: As mentioned above, all figures are converted to high-resolution PDF vector files (≥300 DPI). All graphical elements, labels and annotations are optimized to ensure clarity.

Tables: We have standardized all tables in the manuscript in accordance with PLOS ONE table specifications, adjusted column alignment, line styles and typography, optimized the layout of ablation experiment tables and comparison tables, and made the tables neat, standardized and easy to read.

**Comment 2**: Incomplete ablation experiments. Only single-module and full-module combinations were tested; pairwise combination of two modules is missing.

Response:

We fully supplement the incomplete ablation experiments as required:

Add dual-module combined groups: We supplemented three pairwise combination experimental groups (YOLOv11+RGBT+PSConv, YOLOv11+RGBT+P6, YOLOv11+PSConv+P6) in Table 1 of ablation experiments, and updated all corresponding index data. The revised table covers single-module, dual-module and triple-module all combination modes.

Supplement result analysis: We added detailed analytical descriptions for the three dual-module groups in Section 4.4 Ablation Experiment, and also updated the analysis of corresponding PR curves in Figure 5, discussing the synergistic effect between every two modules.

The revised ablation experiment tables, textual analysis and PR curve explanations have all been updated in the manuscript.

**Part 4 Additional Explanation**

All revisions involved in this manuscript are marked with track changes in the attached Revised Manuscript with Track Changes file for your inspection.

The clean version of the revised manuscript, high-resolution PDF figures, complete source code and supplementary experimental data are all uploaded as required.

We have carefully checked the full text for grammatical errors, spelling mistakes and sentence fluency, and the whole manuscript conforms to standard academic English writing specifications.

Once again, we thank the academic editor and reviewers for your rigorous review and valuable suggestions. Your comments have greatly helped us improve the quality of this manuscript. If you have any further questions or revision requirements, please feel free to contact us. We will actively cooperate and make timely adjustments.

Sincerely,

Corresponding Author : Yangyang Shi

School of Journalism and Communication, Xiamen University

Xiamen, Fujian, China

Email: syy9997@163.com
