# Supplementary material for: An RPP-YOLOv11 model for road crack detection
Source: PLoS One. 2026 Aug 6;21(8):e0355111. doi: 10.1371/journal.pone.0355111 (PMC13446698; doi:10.1371/journal.pone.0355111)
Supplement: Peer review history

# Peer review: Decision Letter 0

**DOI:** 10.1371/journal.pone.0355111.r001

**Authors:** Ardashir Mohammadzadeh

3 May 2026

PONE-D-26-14112

An RPP-YOLOv11 Model for Road Crack Detection

PLOS One

Dear Dr. Shi,

Thank you for submitting your manuscript to PLOS ONE. After careful consideration, we feel that it has merit but does not fully meet PLOS ONE’s publication criteria as it currently stands. Therefore, we invite you to submit a revised version of the manuscript that addresses the points raised during the review process.

Please submit your revised manuscript by Jun 17 2026 11:59PM. If you will need more time than this to complete your revisions, please reply to this message or contact the journal office at plosone@plos.org. When you're ready to submit your revision, log on to https://www.editorialmanager.com/pone/ and select the 'Submissions Needing Revision' folder to locate your manuscript file.

Please include the following items when submitting your revised manuscript:

- A letter that responds to each point raised by the academic editor and reviewer(s). You should upload this letter as a separate file labeled 'Response to Reviewers'.
- A marked-up copy of your manuscript that highlights changes made to the original version. You should upload this as a separate file labeled 'Revised Manuscript with Track Changes'.
- An unmarked version of your revised paper without tracked changes. You should upload this as a separate file labeled 'Manuscript'.

If you would like to make changes to your financial disclosure, please include your updated statement in your cover letter. Guidelines for resubmitting your figure files are available below the reviewer comments at the end of this letter.

If applicable, we recommend that you deposit your laboratory protocols in protocols.io to enhance the reproducibility of your results. Protocols.io assigns your protocol its own identifier (DOI) so that it can be cited independently in the future. For instructions see: https://journals.plos.org/plosone/s/submission-guidelines#loc-laboratory-protocols. Additionally, PLOS ONE offers an option for publishing peer-reviewed Lab Protocol articles, which describe protocols hosted on protocols.io. Read more information on sharing protocols at https://plos.org/protocols?utm_medium=editorial-email&utm_source=authorletters&utm_campaign=protocols.

As the corresponding author, your ORCID iD is verified in the submission system and will appear in the published article. PLOS supports the use of ORCID, and we encourage all coauthors to register for an ORCID iD and use it as well. Please encourage your coauthors to verify their ORCID iD within the submission system before final acceptance, as unverified ORCID iDs will not appear in the published article. *Only*  the individual author can complete the verification step; PLOS staff *cannot*  verify ORCID iDs on behalf of authors.

We look forward to receiving your revised manuscript.

Kind regards,

Ardashir Mohammadzadeh, Phd

Academic Editor

PLOS One

Journal Requirements:

When submitting your revision, we need you to address these additional requirements.

1. Please ensure that your manuscript meets PLOS ONE's style requirements, including those for file naming. The PLOS ONE style templates can be found at

https://journals.plos.org/plosone/s/file?id=wjVg/PLOSOne_formatting_sample_main_body.pdf and

https://journals.plos.org/plosone/s/file?id=ba62/PLOSOne_formatting_sample_title_authors_affiliations.pdf

2. Please note that PLOS One has specific guidelines on code sharing for submissions in which author-generated code underpins the findings in the manuscript. In these cases, we expect all author-generated code to be made available without restrictions upon publication of the work. Please review our guidelines at https://journals.plos.org/plosone/s/materials-and-software-sharing#loc-sharing-code and ensure that your code is shared in a way that follows best practice and facilitates reproducibility and reuse.

3. Thank you for stating the following in the Acknowledgments Section of your manuscript: [This research was funded by the Technology Development Cooperation Project between Shanxi Steel Structure Science & Industry Co., Ltd. and North University of China (Development of Digital Twin Intelligent Inspection System for Building Steel Components, Contract No.: 20260320161524).]

We note that you have provided funding information that is not currently declared in your Funding Statement. However, funding information should not appear in the Acknowledgments section or other areas of your manuscript. We will only publish funding information present in the Funding Statement section of the online submission form.

Please remove any funding-related text from the manuscript and let us know how you would like to update your Funding Statement. Currently, your Funding Statement reads as follows: “The authors received no specific funding for this work.”

Please include your amended statements within your cover letter; we will change the online submission form on your behalf.

4. Thank you for stating the following in the Financial Disclosure section: [The authors received no specific funding for this work].

We note that one or more of the authors are employed by a commercial company: [Shanxi Steel Structure Science & Industry Co., Ltd ]

1. Please provide an amended Funding Statement declaring this commercial affiliation, as well as a statement regarding the Role of Funders in your study. If the funding organization did not play a role in the study design, data collection and analysis, decision to publish, or preparation of the manuscript and only provided financial support in the form of authors' salaries and/or research materials, please review your statements relating to the author contributions, and ensure you have specifically and accurately indicated the role(s) that these authors had in your study. You can update author roles in the Author Contributions section of the online submission form.

Please also include the following statement within your amended Funding Statement.

“The funder provided support in the form of salaries for authors [insert relevant initials], but did not have any additional role in the study design, data collection and analysis, decision to publish, or preparation of the manuscript. The specific roles of these authors are articulated in the ‘author contributions’ section.”

If your commercial affiliation did play a role in your study, please state and explain this role within your updated Funding Statement.

2. Please also provide an updated Competing Interests Statement declaring this commercial affiliation along with any other relevant declarations relating to employment, consultancy, patents, products in development, or marketed products, etc.

Within your Competing Interests Statement, please confirm that this commercial affiliation does not alter your adherence to all PLOS ONE policies on sharing data and materials by including the following statement: ""This does not alter our adherence to  PLOS ONE policies on sharing data and materials.” (as detailed online in our guide for authors http://journals.plos.org/plosone/s/competing-interests) . If this adherence statement is not accurate and  there are restrictions on sharing of data and/or materials, please state these. Please note that we cannot proceed with consideration of your article until this information has been declared.

Please include both an updated Funding Statement and Competing Interests Statement in your cover letter. We will change the online submission form on your behalf.

5. We note that your Data Availability Statement is currently as follows: [All relevant data are within the manuscript and its Supporting Information files]

Please confirm at this time whether or not your submission contains all raw data required to replicate the results of your study. Authors must share the “minimal data set” for their submission. PLOS defines the minimal data set to consist of the data required to replicate all study findings reported in the article, as well as related metadata and methods (https://journals.plos.org/plosone/s/data-availability#loc-minimal-data-set-definition).

For example, authors should submit the following data:

- The values behind the means, standard deviations and other measures reported;

- The values used to build graphs;

- The points extracted from images for analysis.

Authors do not need to submit their entire data set if only a portion of the data was used in the reported study.

If your submission does not contain these data, please either upload them as Supporting Information files or deposit them to a stable, public repository and provide us with the relevant URLs, DOIs, or accession numbers. For a list of recommended repositories, please see https://journals.plos.org/plosone/s/recommended-repositories.

If there are ethical or legal restrictions on sharing a de-identified data set, please explain them in detail (e.g., data contain potentially sensitive information, data are owned by a third-party organization, etc.) and who has imposed them (e.g., an ethics committee). Please also provide contact information for a data access committee, ethics committee, or other institutional body to which data requests may be sent. If data are owned by a third party, please indicate how others may request data access.

6. We note that Figures 1, 7 and 11 in your submission contain copyrighted images. All PLOS content is published under the Creative Commons Attribution License (CC BY 4.0), which means that the manuscript, images, and Supporting Information files will be freely available online, and any third party is permitted to access, download, copy, distribute, and use these materials in any way, even commercially, with proper attribution. For more information, see our copyright guidelines: http://journals.plos.org/plosone/s/licenses-and-copyright.

We require you to either (1) present written permission from the copyright holder to publish these figures specifically under the CC BY 4.0 license, or (2) remove the figures from your submission:

1. You may seek permission from the original copyright holder of Figures 1, 7 and 11 to publish the content specifically under the CC BY 4.0 license.

We recommend that you contact the original copyright holder with the Content Permission Form (http://journals.plos.org/plosone/s/file?id=7c09/content-permission-form.pdf) and the following text:

“I request permission for the open-access journal PLOS ONE to publish XXX under the Creative Commons Attribution License (CCAL) CC BY 4.0 (http://creativecommons.org/licenses/by/4.0/). Please be aware that this license allows unrestricted use and distribution, even commercially, by third parties. Please reply and provide explicit written permission to publish XXX under a CC BY license and complete the attached form.”

Please upload the completed Content Permission Form or other proof of granted permissions as an "Other" file with your submission.

In the figure caption of the copyrighted figure, please include the following text: “Reprinted from [ref] under a CC BY license, with permission from [name of publisher], original copyright [original copyright year].”

2. If you are unable to obtain permission from the original copyright holder to publish these figures under the CC BY 4.0 license or if the copyright holder’s requirements are incompatible with the CC BY 4.0 license, please either i) remove the figure or ii) supply a replacement figure that complies with the CC BY 4.0 license. Please check copyright information on all replacement figures and update the figure caption with source information. If applicable, please specify in the figure caption text when a figure is similar but not identical to the original image and is therefore for illustrative purposes only.

7. If the reviewer comments include a recommendation to cite specific previously published works, please review and evaluate these publications to determine whether they are relevant and should be cited. There is no requirement to cite these works unless the editor has indicated otherwise.

[Note: HTML markup is below. Please do not edit.]

Reviewers' comments:

Reviewer's Responses to Questions

**Comments to the Author**

1. Is the manuscript technically sound, and do the data support the conclusions?

The manuscript must describe a technically sound piece of scientific research with data that supports the conclusions. Experiments must have been conducted rigorously, with appropriate controls, replication, and sample sizes. The conclusions must be drawn appropriately based on the data presented.

Reviewer #1: Yes

Reviewer #2: Yes

**********

2. Has the statistical analysis been performed appropriately and rigorously?

Reviewer #1: Yes

Reviewer #2: Yes

**********

3. Have the authors made all data underlying the findings in their manuscript fully available?

The PLOS Data policy requires authors to make all data underlying the findings described in their manuscript fully available without restriction, with rare exception (please refer to the Data Availability Statement in the manuscript PDF file). The data should be provided as part of the manuscript or its supporting information, or deposited to a public repository. For example, in addition to summary statistics, the data points behind means, medians and variance measures should be available. If there are restrictions on publicly sharing data—e.g. participant privacy or use of data from a third party—those must be specified.

Reviewer #1: Yes

Reviewer #2: No

**********

4. Is the manuscript presented in an intelligible fashion and written in standard English?

PLOS ONE does not copyedit accepted manuscripts, so the language in submitted articles must be clear, correct, and unambiguous. Any typographical or grammatical errors should be corrected at revision, so please note any specific errors here.

Reviewer #1: Yes

Reviewer #2: Yes

**********

5. Review Comments to the Author

Please use the space provided to explain your answers to the questions above. You may also include additional comments for the author, including concerns about dual publication, research ethics, or publication ethics. (Please upload your review as an attachment if it exceeds 20,000 characters)

Reviewer #1: This study addresses the challenge of accurate and efficient road crack detection in complex environments (e.g., low light, shadows, haze) and across varying scales. Building upon the YOLOv11 architecture, the authors aim to enhance feature extraction for fine cracks, global perception of large-scale cracks, and robustness under diverse lighting conditions. The proposed approach is well-motivated by identified limitations in existing methods, and the integration of RGBT fusion, PSConv, and multi-scale detection is coherent. The experimental design is systematic, including ablation studies, comparisons with baselines and state-of-the-art models, and extension experiments. Results demonstrate improvements over YOLOv11 and other benchmarks across key metrics (precision, recall, mAP@0.5, mAP@0.5:0.95), supporting the authors' claims. However, revisions are required to address presentation issues and clarify specific points before acceptance.

*Condense the overview of YOLOv1-v8 in Section 2.1. Focus more detail on YOLOv11 and its specific improvements relevant to this work (e.g., architecture optimizations), and explicitly compare/contrast the proposed RPP-YOLOv11 with closely related models like YOLOv11-DCFNet and YOLOv11-RGBT, highlighting the novelty and differences of the current approach.

*Provide high-resolution versions of ALL figures. Figures 1, 2, 3, and 4 are essential for understanding the model and must be legible. Use vector graphics (PDF, SVG) or high-resolution raster images (minimum 300 DPI). Ensure all text labels, arrows, and diagram elements are crisp and clear both on screen and in print.

*In Section 3.1 (RGBT Fusion), briefly explain the function of the "Silence" and "SilenceChannel" modules. What specific operation do they perform on the 4-channel input to make it compatible with the subsequent backbone network designed for 3 channels? A sentence or two clarifying their role would significantly improve readability.

Reviewer #2: This article focuses on road crack detection and proposes RPP-YOLOv11 based on YOLOv11. The core changes include: early fusion of RGB and thermal infrared information to form 4-channel inputs, introduction of PSConv to replace some standard convolutions, and addition of P6 detection layer to construct a P3-P6 four scale detection framework. The author reported significant improvement in YOLOv11 compared to baseline on RDD2022, and provided ablation experiments, comparisons with YOLOv5/8/10/11, and cross dataset testing on UAV-PDD2023. The overall goal is clear, the method chain is complete, and the paper attempts to simultaneously address issues such as adaptability to complex environments, fine-grained crack characterization, and insufficient receptive field for large-scale cracks.

Strength:

The topic of the article has practical significance. Road crack detection itself is a problem with clear engineering requirements. The author organizes the problem around three pain points: complex lighting, small and slender targets, and large-scale crack missed detection. The problem definition is reasonable. The method design also has a certain degree of intuitiveness: RGBT early fusion, PSConv, and P6 layers correspond to environmental robustness, local detail modeling, and large-scale target coverage, respectively, with relatively clear module responsibilities. The experimental part at least includes single module ablation, comprehensive ablation, comparison of mainstream YOLO series, and cross dataset testing, indicating that the author consciously verifies the effectiveness of the scheme from multiple perspectives. Based on the numbers provided in the manuscript, the complete model is relatively better than YOLOv11 mAP@0.5 The mAP@0.5 0.95, with significant improvements in precision and recall, while the parameter count remains relatively small, which is attractive for engineering deployment.

However, there are some issues that need to be addressed as follows.

1. Format: The clarity of all images is severely lacking, and the table design is not standardized and aesthetically pleasing.

2. The ablation experiment is not yet complete. The author only conducted single module and full module combinations, but did not analyze pairwise combinations between modules, nor did they report statistical stability, such as running mean/variance multiple times or different random seed results.

**********

6. PLOS authors have the option to publish the peer review history of their article (what does this mean?). If published, this will include your full peer review and any attached files.

If you choose “no”, your identity will remain anonymous but your review may still be made public.

**Do you want your identity to be public for this peer review?**  For information about this choice, including consent withdrawal, please see our Privacy Policy.

Reviewer #1: No

Reviewer #2: **Yes:**  Guohua Lv

**********

[NOTE: If reviewer comments were submitted as an attachment file, they will be attached to this email and accessible via the submission site. Please log into your account, locate the manuscript record, and check for the action link "View Attachments". If this link does not appear, there are no attachment files.]

To ensure your figures meet our technical requirements, please review our figure guidelines: https://journals.plos.org/plosone/s/figures

You may also use PLOS’s free figure tool, NAAS, to help you prepare publication quality figures: https://journals.plos.org/plosone/s/figures#loc-tools-for-figure-preparation.

NAAS will assess whether your figures meet our technical requirements by comparing each figure against our figure specifications.

# Author comment: Author response to Decision Letter 1

**DOI:** 10.1371/journal.pone.0355111.r002

4 Jul 2026

Dear Academic Editor Dr. Ardahir Mohammadzadeh and respected Reviewers,

We sincerely appreciate the valuable comments, detailed suggestions and constructive revisions from the academic editor and two reviewers. We have carefully studied all the feedback, revised the manuscript item by item strictly following your requirements, supplemented missing experimental content, adjusted format specifications, optimized figures and relevant statements. Below is our point-by-point response to all questions and requirements. The revised manuscript with track changes, clean manuscript and all supplementary files are submitted together as required.

Part 1 Response to General Requirements from PLOS ONE Academic Editor

1. Manuscript Format Compliance

We have fully revised the manuscript in accordance with the PLOS ONE official formatting templates (main body and title & author affiliation templates). All layout, font, paragraph format, citation style and table/figure caption rules have been adjusted to meet the journal’s standard requirements.

2. Code Sharing Policy

Following PLOS ONE’s code sharing and reproducibility guidelines, we have uploaded the complete source code of the proposed RPP-YOLOv11 model to a public open-source repository. The access link is: https://github.com/wolfololo/RPP-YOLOv11. The code is fully open for peer review, experimental reproduction and academic reuse, complying with the journal’s code sharing best practices.

3. Funding Statement Revision

As required, we have removed all funding-related content from the Acknowledgments section of the manuscript. The revised formal funding statement is provided below for updating in the online submission system:

This research was funded by the Technology Development Cooperation Project between Shanxi Steel Structure Science & Industry Co., Ltd. and North University of China (Development of Digital Twin Intelligent Inspection System for Building Steel Components, Contract No.: 20260320161524). The funders provided salary support for the authors affiliated with Shanxi Steel Structure Science & Industry Co., Ltd., but had no role in the study design, data collection and analysis, decision to publish, or preparation of the manuscript. The specific contributions of all authors are detailed in the Author Contributions section.

4. Competing Interests & Commercial Relationship Statement

Some authors are employed by Shanxi Steel Structure Science & Industry Co., Ltd. We hereby submit the updated competing interests statement as follows:

One or more authors are employed by Shanxi Steel Structure Science & Industry Co., Ltd. This commercial affiliation does not alter our adherence to all PLOS ONE policies on sharing data and materials. There are no patents, products in development or marketed products related to this work to declare.

5. Data Availability & Minimum Dataset Confirmation

The public datasets for road defect detection used in this study can all be obtained through official designated channels, with the specific acquisition methods as follows:

RDD2022 Dataset�https://github.com/sekilab/RoadDamageDetector

UAV-PDD2023 Dataset�https://doi.org/10.1016/j.dib.2023.109692

The source code of the proposed RPP-YOLOv11 model is available at https://github.com/wolfololo/RPP-YOLOv11 for peer review and experimental reproduction.All minimal datasets required to replicate all experimental results of this study, including model evaluation metrics, training records, raw curve images, confusion matrices and detection visualization figures, are provided within the manuscript’s Supporting Information supplementary file.

6. Copyright Issues of Figures

We hereby clarify the copyright of all figures in the manuscript:

Figure 1: model structure diagrams is independently drawn by the authors, with no external copyright restrictions.

Figure 7, Figure 11: These experimental result comparison graphs are generated from our self-conducted road crack detection experiments, using original data and images collected in this study. There is no third-party copyright involved.

All figures have been converted to PDF vector format with a resolution higher than 300 DPI as required. The blurriness in the previous submission was caused by platform automatic compression. The newly uploaded high-resolution PDF figures ensure all text, labels, arrows and graphical elements are clear for both screen display and printing. No copyright permission from third parties is needed for all figures in this manuscript.

Part 2 Response to Comments from Reviewer 1

Comment 1: Revise Section 2.1: Shorten the overview of YOLOv1–v8, elaborate on YOLOv11’s improvements related to this work, and clearly compare RPP-YOLOv11 with YOLOv11-DCFNet and YOLOv11-RGBT to highlight novelty.

Response:

We have comprehensively revised Section 2.1 YOLO Series Models as required:

We greatly condensed the introductory content of YOLOv1 to YOLOv8, retaining only core functional characteristics closely related to pavement detection and removing redundant iterative descriptions.

We supplemented detailed explanations of YOLOv11’s native architectural optimizations, including lightweight C3k2 modules, SPPELAN neck and decoupled detection heads, and analyzed the defects of the original YOLOv11 in processing 4-channel RGBT multimodal data.

We made a targeted comparative analysis between our RPP-YOLOv11 and two closely related variants (YOLOv11-DCFNet, YOLOv11-RGBT). We pointed out the shortcomings of late fusion and simple channel compression in the existing methods, and highlighted the novelty of our cascaded Silence & SilenceChannel modules, PSConv and P6 detection layer. The revised content has been marked in the track-changes manuscript.

Comment 2: Provide high-resolution figures (Figure 1, 2, 3, 4) with clear labels and elements (minimum 300 DPI, vector format such as PDF/SVG is preferred).

Response:

We have reprocessed all key figures. All figures are exported as PDF vector files with a resolution of 300 DPI or above. The blurry problem in the last submission was caused by the platform’s automatic compression of raster images. The newly uploaded PDF figures guarantee that all text labels, arrows, network structures and module details are clearly recognizable on screen and in printed versions.

Comment 3: In Section 3.1 (RGBT fusion), briefly explain the core functions of Silence and SilenceChannel modules, as well as their specific operations for 4-channel input to adapt the 3-channel backbone network, to improve readability.

Response:

We have added targeted descriptions of the two modules in the Input Layer part of Section 3.1 on the basis of the original text:

We briefly stated the core function of Silence and SilenceChannel: solving the channel dimension mismatch between 4-channel RGBT input and the original 3-channel YOLOv11 backbone.

We detailed the specific operations: the Silence module suppresses invalid thermal infrared background noise via soft channel masking on the 4-channel tensor; the SilenceChannel module uses learnable 1×1 convolution to compress 4 channels into standard 3-channel feature maps.

We clarified that this lightweight operation does not modify the pre-trained weights of the backbone network, realizing seamless adaptation. The revised content is concise and compliant with the requirements, and the readability of this section is significantly improved.

Part 3 Response to Comments from Reviewer 2

Comment 1: Poor clarity of all figures and non-standard table layout.

Response:

Figures: As mentioned above, all figures are converted to high-resolution PDF vector files (≥300 DPI). All graphical elements, labels and annotations are optimized to ensure clarity.

Tables: We have standardized all tables in the manuscript in accordance with PLOS ONE table specifications, adjusted column alignment, line styles and typography, optimized the layout of ablation experiment tables and comparison tables, and made the tables neat, standardized and easy to read.

Comment 2: Incomplete ablation experiments. Only single-module and full-module combinations were tested; pairwise combination of two modules is missing.

Response:

We fully supplement the incomplete ablation experiments as required:

Add dual-module combined groups: We supplemented three pairwise combination experimental groups (YOLOv11+RGBT+PSConv, YOLOv11+RGBT+P6, YOLOv11+PSConv+P6) in Table 1 of ablation experiments, and updated all corresponding index data. The revised table covers single-module, dual-module and triple-module all combination modes.

Supplement result analysis: We added detailed analytical descriptions for the three dual-module groups in Section 4.4 Ablation Experiment, and also updated the analysis of corresponding PR curves in Figure 5, discussing the synergistic effect between every two modules.

The revised ablation experiment tables, textual analysis and PR curve explanations have all been updated in the manuscript.

Part 4 Additional Explanation

All revisions involved in this manuscript are marked with track changes in the attached Revised Manuscript with Track Changes file for your inspection.

The clean version of the revised manuscript, high-resolution PDF figures, complete source code and supplementary experimental data are all uploaded as required.

We have carefully checked the full text for grammatical errors, spelling mistakes and sentence fluency, and the whole manuscript conforms to standard academic English writing specifications.

Once again, we thank the academic editor and reviewers for your rigorous review and valuable suggestions. Your comments have greatly helped us improve the quality of this manuscript. If you have any further questions or revision requirements, please feel free to contact us. We will actively cooperate and make timely adjustments.

Attachment Submitted filename: Response to Reviewers.docx

# Peer review: Decision Letter 1

**DOI:** 10.1371/journal.pone.0355111.r003

**Authors:** Gaurav Arora

17 Jul 2026

An RPP-YOLOv11 Model for Road Crack Detection

PONE-D-26-14112R1

Dear Dr. Shi,

We’re pleased to inform you that your manuscript has been judged scientifically suitable for publication and will be formally accepted for publication once it meets all outstanding technical requirements.

Within one week, you’ll receive an e-mail detailing the required amendments. When these have been addressed, you’ll receive a formal acceptance letter and your manuscript will be scheduled for publication.

An invoice will be generated when your article is formally accepted. Please note, if your institution has a publishing partnership with PLOS and your article meets the relevant criteria, all or part of your publication costs will be covered. Please make sure your user information is up-to-date by logging into Editorial Manager at Editorial Manager® and clicking the ‘Update My Information' link at the top of the page. For questions related to billing, please contact billing support.

If your institution or institutions have a press office, please notify them about your upcoming paper to help maximize its impact. If they’ll be preparing press materials, please inform our press team as soon as possible -- no later than 48 hours after receiving the formal acceptance. Your manuscript will remain under strict press embargo until 2 pm Eastern Time on the date of publication. For more information, please contact onepress@plos.org.

Kind regards,

Academic Editor

PLOS One

Additional Editor Comments (optional):

The reviewers have no more questions.

Reviewers' comments:

Reviewer's Responses to Questions

**Comments to the Author**

1. If the authors have adequately addressed your comments raised in a previous round of review and you feel that this manuscript is now acceptable for publication, you may indicate that here to bypass the “Comments to the Author” section, enter your conflict of interest statement in the “Confidential to Editor” section, and submit your "Accept" recommendation.

Reviewer #1: (No Response)

Reviewer #2: All comments have been addressed

**********

2. Is the manuscript technically sound, and do the data support the conclusions?

The manuscript must describe a technically sound piece of scientific research with data that supports the conclusions. Experiments must have been conducted rigorously, with appropriate controls, replication, and sample sizes. The conclusions must be drawn appropriately based on the data presented.

Reviewer #1: (No Response)

Reviewer #2: Yes

**********

3. Has the statistical analysis been performed appropriately and rigorously?

Reviewer #1: (No Response)

Reviewer #2: Yes

**********

4. Have the authors made all data underlying the findings in their manuscript fully available?

The PLOS Data policy requires authors to make all data underlying the findings described in their manuscript fully available without restriction, with rare exception (please refer to the Data Availability Statement in the manuscript PDF file). The data should be provided as part of the manuscript or its supporting information, or deposited to a public repository. For example, in addition to summary statistics, the data points behind means, medians and variance measures should be available. If there are restrictions on publicly sharing data—e.g. participant privacy or use of data from a third party—those must be specified.

Reviewer #1: (No Response)

Reviewer #2: Yes

**********

5. Is the manuscript presented in an intelligible fashion and written in standard English?

PLOS ONE does not copyedit accepted manuscripts, so the language in submitted articles must be clear, correct, and unambiguous. Any typographical or grammatical errors should be corrected at revision, so please note any specific errors here.

Reviewer #1: (No Response)

Reviewer #2: Yes

**********

6. Review Comments to the Author

Please use the space provided to explain your answers to the questions above. You may also include additional comments for the author, including concerns about dual publication, research ethics, or publication ethics. (Please upload your review as an attachment if it exceeds 20,000 characters)

Reviewer #1: (No Response)

Reviewer #2: (No Response)

**********

7. PLOS authors have the option to publish the peer review history of their article (what does this mean?). If published, this will include your full peer review and any attached files.

If you choose “no”, your identity will remain anonymous but your review may still be made public.

**Do you want your identity to be public for this peer review?**  For information about this choice, including consent withdrawal, please see our Privacy Policy.

Reviewer #1: No

Reviewer #2: **Yes:**  Guohua Lv

**********

# Editor report: Acceptance letter

**DOI:** 10.1371/journal.pone.0355111.r004

**Authors:** Gaurav Arora

PONE-D-26-14112R1

PLOS One

Dear Dr. Shi,

I'm pleased to inform you that your manuscript has been deemed suitable for publication in PLOS One. Congratulations! Your manuscript is now being handed over to our production team.

At this stage, our production department will prepare your paper for publication. This includes ensuring the following:

* All references, tables, and figures are properly cited

* All relevant supporting information is included in the manuscript submission,

* There are no issues that prevent the paper from being properly typeset

You will receive further instructions from the production team, including instructions on how to review your proof when it is ready. Please keep in mind that we are working through a large volume of accepted articles, so please give us a few days to review your paper and let you know the next and final steps.

Lastly, if your institution or institutions have a press office, please let them know about your upcoming paper now to help maximize its impact. If they'll be preparing press materials, please inform our press team within the next 48 hours. Your manuscript will remain under strict press embargo until 2 pm Eastern Time on the date of publication. For more information, please contact onepress@plos.org.

You will receive an invoice from PLOS for your publication fee after your manuscript has reached the completed accept phase. If you receive an email requesting payment before acceptance or for any other service, this may be a phishing scheme. Learn how to identify phishing emails and protect your accounts at https://explore.plos.org/phishing.

If we can help with anything else, please email us at customercare@plos.org.

Thank you for submitting your work to PLOS ONE and supporting open access.

Kind regards,

PLOS ONE Editorial Office Staff

on behalf of

Dr. Gaurav Arora

Academic Editor

PLOS One
